# Multi-network collaborative lift-drag ratio prediction and airfoil optimization based on residual network and generative adversarial network

**DOI:** 10.3389/fbioe.2022.927064

**Published:** 2022-09-06

**Authors:** Xiaoyu Zhao, Weiguo Wu, Wei Chen, Yongshui Lin, Jiangcen Ke

**Affiliations:** ^1^ Hubei Key Laboratory of Theory and Application of Advanced Materials Mechanics, Department of Engineering Structure and Mechanics, Wuhan University of Technology, Wuhan, China; ^2^ Green and Smart River-Sea-Going Ship, Cruise and Yacht Research Center, Wuhan, China; ^3^ School of Transportation and Logistics Engineering, Wuhan University of Technology, Wuhan, China

**Keywords:** deep learning, airfoil, particle swam optimisation, random, parameter optimizatioin

## Abstract

As compared with the computational fluid dynamics(CFD), the airfoil optimization based on deep learning significantly reduces the computational cost. In the airfoil optimization based on deep learning, due to the uncertainty in the neural network, the optimization results deviate from the true value. In this work, a multi-network collaborative lift-to-drag ratio prediction model is constructed based on ResNet and penalty functions. Latin supersampling is used to select four angles of attack in the range of 2°–10° with significant uncertainty to limit the prediction error. Moreover, the random drift particle swarm optimization (RDPSO) algorithm is used to control the prediction error. The experimental results show that multi-network collaboration significantly reduces the error in the optimization results. As compared with the optimization based on a single network, the maximum error of multi-network coordination in single angle of attack optimization reduces by 16.0%. Consequently, this improves the reliability of airfoil optimization based on deep learning.

## Introduction

The airfoils are widely used in aerospace, marine engineering, new energy exploration, and other fields. In the field of new energy, in addition to environmental benefits, the wind power has the potential to make a significant contribution to the growing energy demand Office (2004). The airfoil structure significantly influences the energy efficiency of wind power generation Sharma, Gupta, Pandey, Sharma, & Mishra (2021). Due to the high cost of CFD calculation Koziel & Leifsson (2013), - The aerodynamic performance optimization Bedon et al. (2016); Chen and Li (2019); Kallath et al. (2021); Mukesh et al. (2012); Saleem and Kim (2020); Tang et al. (2017) and robustness optimization Reis et al. (2019) of Airfoil Based on CFD are limited. Therefore, optimizing the efficiency of airfoil structures has been a focus of research community.

Improving the optimization algorithm is an important approach for improving the efficiency of airfoilX. Liu et al. (2022a); Liu et al. (2022b). The traditional optimization algorithms include genetic algorithm (GA) Bagley (1967), simulated annealing algorithm (SA) Bangert (2012), and particle swarm optimization (PSO) Kennedy and Eberhart, (1995); Wu et al. (2022). Ram et al. combined the CFD with genetic algorithms to optimize the blades of wind turbine under low Reynolds number with multiple objectives Ram et al. (2013). This improved the performance of airfoil under adverse conditions. Mukesh et al. studied the performance of different optimization algorithms, such as GA and PSO based on CFD. The authors concluded that as compared with other methods, the PSO method has a greater ability to find the global optimal value Mukesh et al. (2012). Chen et al. further fused the GA and PSO to optimize the NACA0018 wing sail and obtained a larger thrust than the original wing Chen and Li (2019). The random drift particle swarm optimization (RDPSO) simulates the random thermal motion of particles by adding a temperature term to the PSO, which improves the global search capability of particles Sun, Wu, Palade, Fang, & Shi (2015). In addition, some scholars have tried to use deep learning to improve the numerical solution of <u>Pawar et al. (2019); Yin et al. (2020).

Using a proxy model is another effective way to enhance the efficiency of airfoil. The common proxy models include Kriging He et al. (2020); LI and PAN (2019); Bu et al. (2020) and the response surface mo Ahn et al. (2001); Sun, (2011); Vavalle and Qin (2012). Recently, deep learning methods have advanced rapidly Huang et al. (2022);Huang et al. (2021); Jiang et al. (2021a); Jiang et al. (2021b). The convolutional neural network (CNN) Fukushima (1980) has achieved good results in various tasks, such as image classification >Gong & Cao (2014);Mahapatra et al. (2016) and prediction <u>Kouhi & Keynia (2013);Mozo et al. (2018). Yu et al. used an improved CNN for predicting airfoil lift coefficients. Forecast so that the root mean square error is less than $3.0\times 10^{-4}$ Yu, Xie, & Wang (2020). In addition to CNN, other types of networks are also used in airfoil optimization. Haryanto, I et al. used ANN and GA methods to optimize the maximum lift-to-drag ratio of an airfoil with high accuracy Haryanto, Utomo, Sinaga, Rosalia, & Putra (2014). Tao et al. used the deep belief network (DBN) to construct a multi-fidelity proxy model for optimizing the robustness of the airfoil Tao & Sun (2019). Considering the training difficulty of deep networks, He et al. proposed ResNet, which further improved the mapping ability of the neural networks by enabling the network to learn the residuals between layers He, Zhang, Ren, & Sun (2016).

However, the optimization based on deep learning is accompanied by black-box adversarial attacks on the neural networks by optimization algorithms Papernot, Mcdaniel, & Goodfellow (2016). That is, the optimization algorithm only requires the value of the optimization function to be optimal, which may lead the neural network to give false optimal predictions. There are various methods to suppress over-fitting Ying (2019) and improve the robustness of the neural networks. Few researchers have proposed methods to defend against these attacks, such as random input transformation Xie, Wang, Zhang, Ren, & Yuille (2017), random noise Liu et al. (2018), and random pruning Dhillon et al. (2018). These methods have achieved good results in graph classification. However, the prediction of airfoil lift-drag ratio requires the neural network to give accurate prediction values. The disturbance of airfoil geometric parameters affects its aerodynamic parameters, limiting the application of the aforementioned methods in airfoil optimization.

The lift-to-drag ratio is unique for certain airfoils and operating conditions. Therefore, the values predicted by different networks should converge to the true values. When the network gives false predictions due to the uncertainty in the neural network Kendall & Gal (2017), the predicted value of different networks has large differences in probability. Based on this principle, in this work, we use multiple ResNets to collaboratively predict the lift-to-drag ratio of the airfoil. In addition, we combine the RDPSO algorithm for optimizing the airfoil for verifying that the collaborative network is in lift-to-drag ratio.

PARSEC parameterization has been applied in several studies (Mukesh et al., 2012; Da et al., 2017, MANAS S. K., 2008). The research by DA et al. and MANAS is based on the PSO algorithm for airfoil optimization. Based on the PARSEC parameterization method, Mukesh et al. (Mukesh, R et al., 2012) compared the optimization results of the Panel Technique and the PSO algorithm. They concluded that the PSO algorithm could find the optimal solution more effectively.

## Materials and methods

**FIGURE 1:**
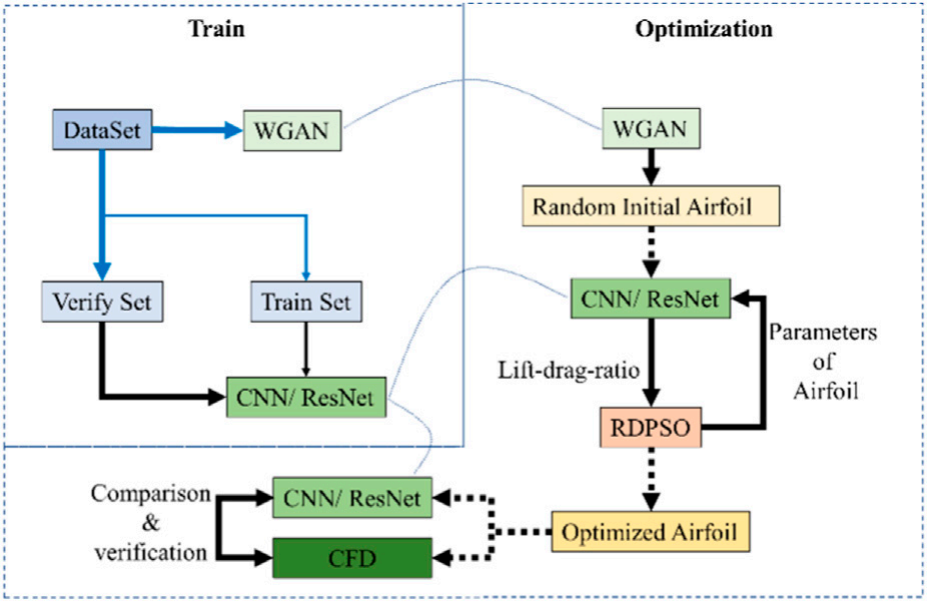
Flow chart of optimization of lift-drag ratio.

The overall process of this paper is shown in the figure below. The GAN network for generating the initial airfoil and the CNN and ResNet for predicting the lift-drag ratio are trained based on the data set. After the GAN generates the initial airfoil, the airfoil is optimized using ResNet and CNN combined with the RDPSO algorithm. Finally, the optimization results are verified by CFD. The optimization process is shown in Figure 1.

### Resnet

The ResNet was proposed by He et al., in 2016 He et al. (2016). It uses shortcut paths in the CNN architecture Fukushima (1980) so that the network can learn the residuals during the training process, thus making the training process easier. The convolutional layers, activation functions, and shortcuts are used to form the blocks in a certain order. Then, these blocks are used to build the entire network.

**FIGURE 2:**
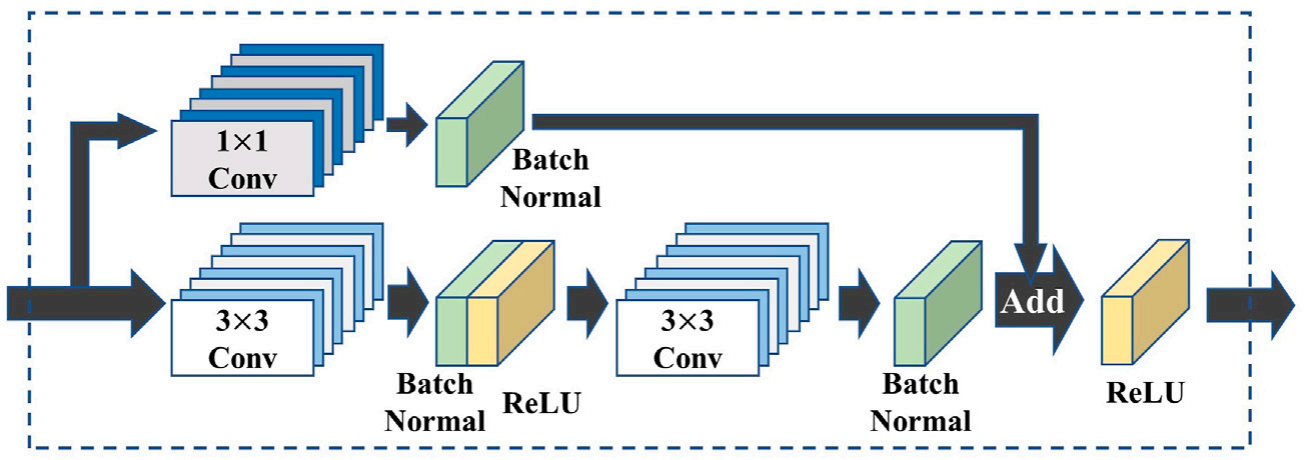
ResBlock structure.

**FIGURE 3:**
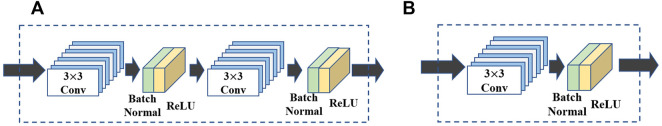
CNN Block structure **(A)** Based on ResBlock and Shortcut bypass deletion **(B)** Typical CNN structure.

The ResNet architecture used in this work is shown in Figure 2. In He et al. (2016), the author uses convolution for down-sampling. In the preliminary tests, the performance of the average pooling layer is slightly better than convolution. Therefore, in this work, we use average pooling for performing down-sampling. We add the dropout Ying (2019) between the last convolutional layer and fully connected (FC) layer to suppress overfitting. In addition, in this work, we use the traditional CNN architecture similar to ResNet (Figure 3A) and a simplified CNN architecture (Figure 3B to build different networks, and compare the prediction capabilities of various networks for airfoil lift-drag ratio.

**FIGURE 4:**
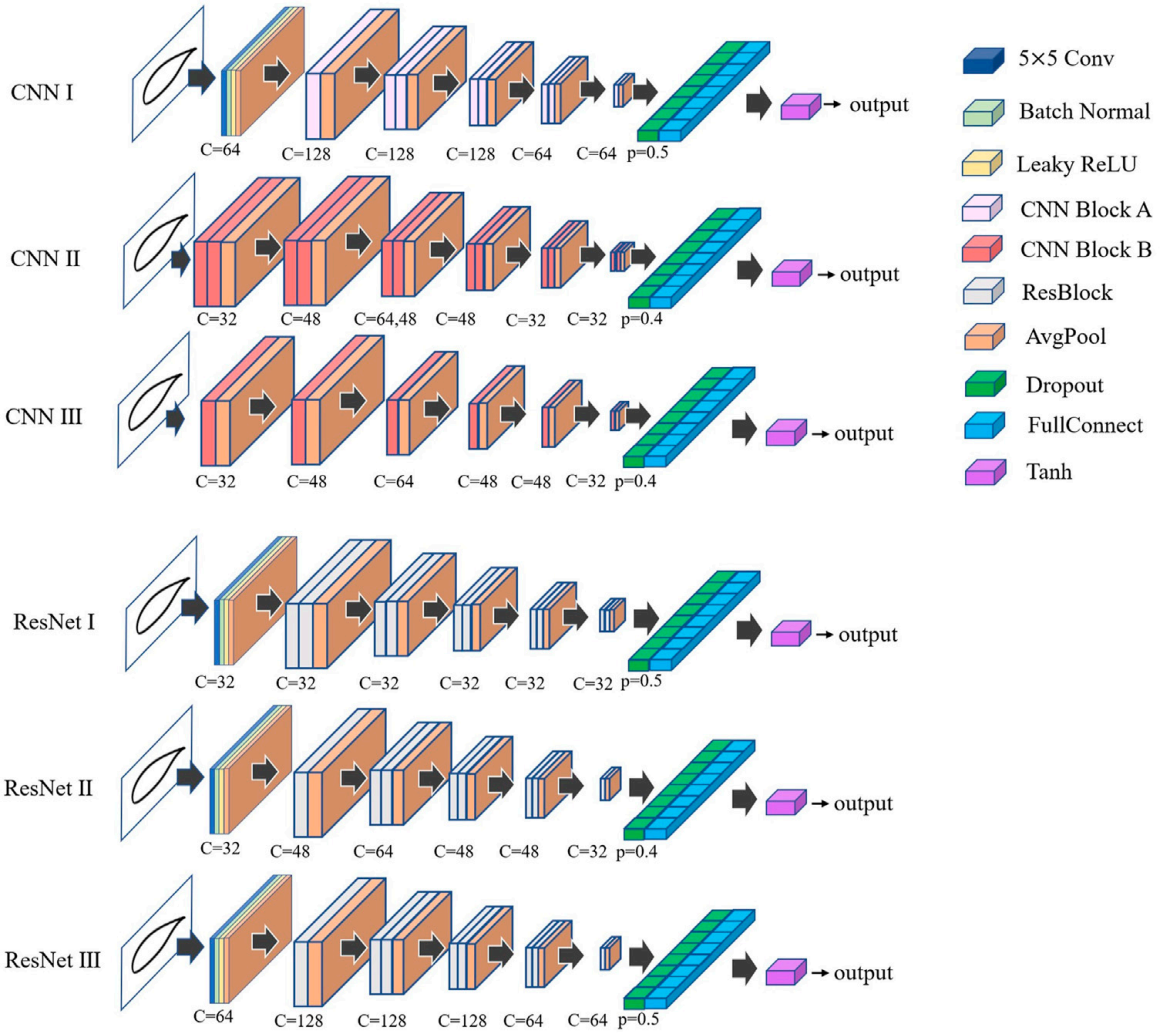
CNN and ResNet structure (when two blocks with the same output channel overlap, Only mark once, such as c = 64. When two blocks with different output channels overlap, mark in order, such as c = 64, 48 means that the output channel of the previous block is 64, and the output channel of the next block is 48)

Based on the three aforementioned types of blocks, in this work, a total of six networks are constructed for training, among which three networks are CNN. As shown in Figure 4, the CNN Ⅰ is composed of CNN Block A, and CNN Ⅱ and CNN Ⅲ are composed of CNN Block B. The other three networks are ResNets, which are composed of ResNet blocks. The input information is a single-channel airfoil geometry. After down-sampling based on convolution, normalization, and pooling, it enters the block for processing. In Figure 4, it is noticeable that the dropout probability of each network is different. This is due to the slight adjustment in the dropout probability of each network during the tests, so that the network is able to reach the lowest possible value of the loss function.

### Airfoil parameterization

The improved geometric parameter (IPG) parameterization method was proposed by Liu et al. This method can decompose the airfoil geometry into the midline and thickness expressions Lu, Huang, Song, & Li (2018) and establish the relationship between the geometric parameters and curve parameters.

In the IPG parameterization, the airfoil centerline is expressed as follows:

$$\begin{array}{c} \{\begin{array}{c} \begin{array}{c} x_{c}=3c_{1}k{\left(1-k\right)}^{2}+3c_{2}\left(1-k\right)k^{2}+k^{3}\\ y_{c}=3c_{3}k{\left(1-k\right)}^{2}+2c_{4}\left(1-k\right)k^{2} \end{array} \end{array} \end{array}$$ (1)

where, K denotes the curve parameter and k∈[0,1] , and $c_{1},c_{2},c_{3},c_{4}$ denote the airfoil design parameters.

The airfoil thickness is expressed as follows:

$$\begin{array}{c} t=t_{1}x^{0.5}+t_{2}x+t_{3}x^{2}+t_{4}x^{4} \end{array}$$ (2)

where, x denotes the abscissa of the airfoil centerline and $x=x_{c}\left(k\right)$ , and $t_{i}\left(i=1,2,3,4,5\right)$ denotes the airfoil design parameter. In order to ensure that the end of the airfoil is closed, $t_{5}=-\sum_{i=1}^{4}t_{i}$ .

### Random drift particle swarm optimization (RDPSO)

The RDPSO is an optimization method Sun et al. (2015) based on PSO Kennedy & Eberhart (1995). For a particle in a dimensional space, the particle coordinate iteration process is expressed as follows:

for n=1,2,3,…,Iterations

for i=1,2,3,…,M

for j=1,2,3,…,N

$$\begin{array}{l} \;\;\;\;\;\;p_{i,n}^{j}=\phi_{i,n}^{j}P_{i,n}^{j}+\left(1-\phi_{i,n}^{j}\right)G_{n}^{j}\\ \;\;\;\;\;\;C_{n}^{j}=\frac{1}{M}\sum_{k=1}^{M}P_{k,n}^{j}\\ \;\;\;\;\;\;V_{i,n+1}^{j}=\alpha |C_{n}^{j}-X_{i,n}^{j}|\varphi_{i,n+1}^{j}+\beta \left(p_{i,n}^{j}-X_{i,n}^{j}\right)\\ \;\;\;\;\;\;X_{i,n+1}^{j}=X_{i,n}^{j}+V_{i,n+1}^{j}\\ \;\;\;\;end\\ \;\;end\\ end \end{array}$$

where, the superscript represents the component of the particles in dimension j and, the subscript *i* denotes the particle number and i=1,2,3,…,M , *n* represents the number of iterations, $P_{i,n}^{j}$ denotes the coordinate of the individual optimal value of the particle *i* in dimension *j* during the first *n* iterations, $\phi_{i,n}^{j}$ denotes a random number distributed between 0 and 1, i.e., $\phi_{i,n}^{j}∼U\backslash \left(0,1\right)$ , $G_{n}^{j}$ denotes the coordinate of the optimal particle in dimension *j* during the first *n* iterations, $C_{n}^{j}$ denotes the average value of the coordinates corresponding to the optimal values of all particles, V denotes the particle velocity, which is the coordinate migration of the particle in this iteration, α represents the temperature factor and 0<α<1 , β denotes the drift factor and 0<β<2 , X denotes the particle coordinate, i.e., the optimization variable, and φ denotes a normally distributed random value, i.e., φ∼N(0,1) .

When the coordinate of a certain particle is randomly selected as ${C^{\prime }}_{n}$ with a probability of 1/M , the expected value of ${C^{\prime }}_{n}^{,j}$ is equivalent to $C_{n}^{j}$ , but the ability of the particle to search for the optimal value can be improved. This method is called RDPSO-Gbest-RP. In this work, we use RDPSO-Gbest-RP for optimization.

### Generative adversarial network (GAN)

**FIGURE 5:**
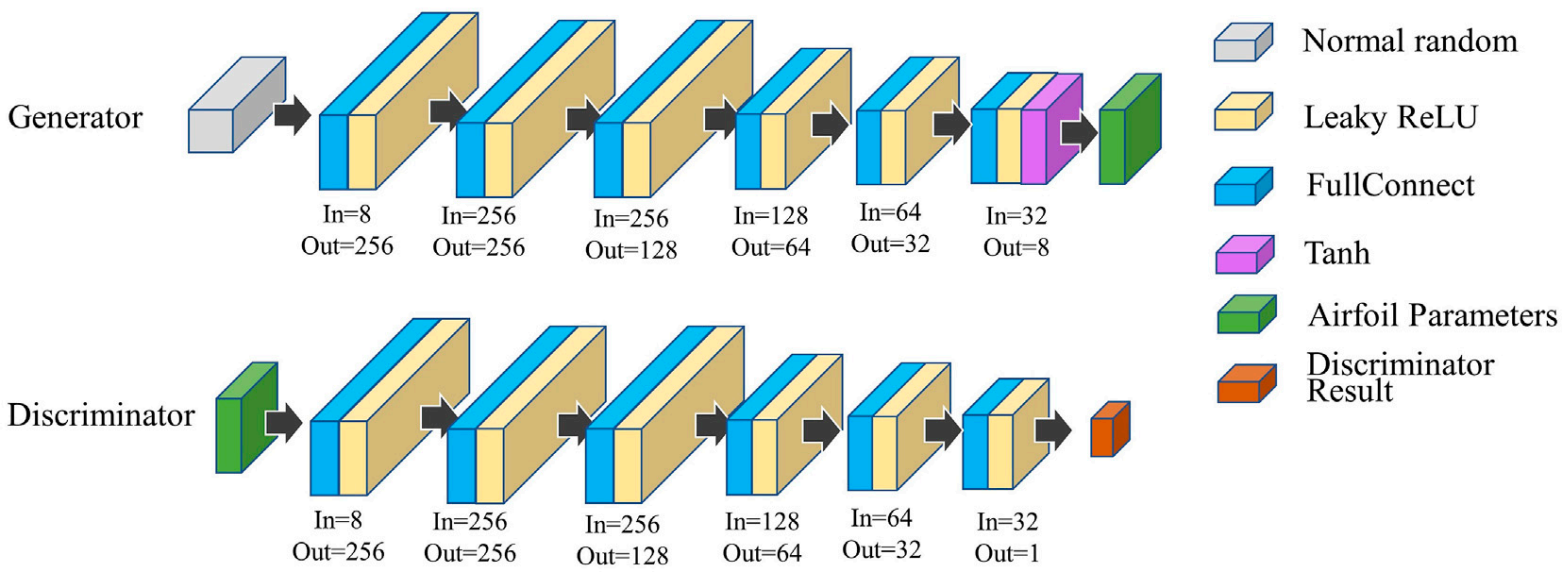
WGAN structure diagram.

The RDPSO method requires that optimization starts from a random number of particles. Usually, the random particles are generated separately in each dimension in the optimized space based on random numbers. As the optimization parameters in the IPG parameterization may not be independent, and the optimization space is irregular, random number generation may cause a large number of particle coordinates corresponding to the airfoil geometry to be abnormal. GAN Goodfellow, (2020) can generate data with the same distribution as the input samples. WGAN-GP Gulrajani, Ahmed, Arjovsky, Dumoulin, & Courville (2017) overcomes the problem of GAN mode collapse. In this work, WGAN-GP is used to generate the random particles to ensure particle randomness and a reasonable range for each parameter. The network architecture is presented in Figure 5.

### Joint network and constraints

This work uses a penalty function for any target angle of attack Deb (2000) to constrain the parameters. The traditional penalty function is mathematically expressed as follows:

$$F_{\alpha }\left(x\right)=f\left(x\right)+\sigma \begin{array}{c} \sum_{i=1}^{n}h_{i}\left(x\right) \end{array},$$ (3)

where f(x) denotes the original optimization objective function, $F_{\alpha }\left(x\right)$ denotes the optimization objective function, σ denotes the penalty factor, and $h_{i}\left(x\right)$ denotes the constraint.

For inequality constraints

$$g_{1}\left(\text{x}\right)\geq g_{1}\begin{array}{c} \left(x_{0}\right) \end{array}$$ (4)

So,

$$h_{1}\left(x\right)=min\left\{0,g_{1}\left(x\right)-g_{1}\left(x_{0}\right)\right\}$$ (5)

When the resistance ratio is predicted for a certain angle of attack, the original optimization objective function is expressed as:

$$\begin{array}{c} f=0.5-0.5\left(wV_{U}+\left(1-w\right)V_{L}\right) \end{array}$$ (6)

The optimization constraint is mathematically expressed as follows:

$$\begin{array}{c} \{\begin{array}{cc} g_{1}=t_{\text{min}}{|}_{0.1<x<0.9} & \geq 0.01\\ g_{2}=\frac{\left|V_{U}-V_{L}\right|}{V_{s,U}} & \leq e_{max}\\ g_{3}=c_{1} & \geq 0.01\\ g_{4}=c_{1}^{2}-c_{1}c_{2}-c_{1}+c_{2}^{2} & <0 \end{array} \end{array}$$ (7)

Among them, $g_{1}$ constrains the minimum thickness of the airfoil, $g_{2}$ constrains the prediction error of the lift-drag ratio, and $g_{3}$ and $g_{4}$ are constraints on the airfoil parameters in the IPG parameterization. The original objective function, $V_{U},V_{L}$ denotes the larger and the smaller output values of the two neural networks, respectively. Since the tan⁡h() activation function is added at the end of the network, and we get −1<V<1 . In (6), w denotes the coefficient that controls the proportion of the larger value to the smaller value.

In the constraint expression, the first item denotes the minimum thickness constraint. The second term represents the error limit, which is used to filter the inconsistent results from the predictions of the two networks and represents the allowable relative error of the two network predictions. It is the maximum value of the lift-to-drag ratio in the sample scaled to the output range of the network. The third item represents the constraint on the parameter. The lower limit of this parameter is presented in Lu et al. (2018). The fourth term is used to prevent the airfoil from curling, when the parameter satisfies (3).

During the optimization process, in addition to the error control of the m target angles of attack, the error penalty should also be performed on the additional n angles of attack, i.e.,

$$\begin{array}{c} F_{net}=\sum_{i=1}^{m}w_{\alpha ,i}F_{\alpha ,i}+\overset{n}{\underset{i=1}{\sum }}g_{2,i} \end{array}$$ (8)

where, $F_{net}$ denotes the value applied to the RDPSO algorithm, m denotes the number of target angles of attack, $w_{\alpha ,i}$ denotes the weight of the *i*th angle of attack, $F_{\alpha ,i}$ denotes the optimized function value of the *i*th angle of attack, n denotes the number of error sampling points selected from the error limit area, and $g_{2,i}$ denotes the value of the penalty term for the *ith* error sampling point.

## Results and discussion

### Network training

**FIGURE 6:**
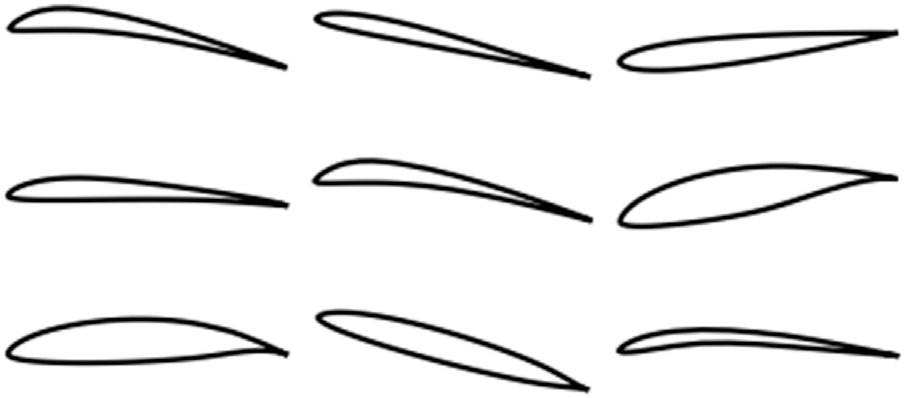
Some airfoil geometry samples in the database.

Airfoiltools Pescador (2016) is a free online airfoil research tool, which currently provides the data of 1,638 airfoils. This tool provides the coordinates of interpolation point of the airfoil, and a list of angles of attack and aerodynamic parameters under the partial Reynolds number. We collected 1,638 airfoils from this website. In order to ensure the quality of the dataset, 1,546 available airfoils are manually screened out, accounting for 94.4% of the total samples. The discarded airfoils include possible coordinate errors, and the data samples where the chord length is significantly smaller than most of the other airfoils. We randomly select 1,233 airfoils for training the network. The remaining data samples are used as an evaluation set. We use the matplotlib library to convert the airfoil into an image with a height of 2 inches, a length of 4 inches, a resolution of 128 × 64, and a line width of 5.0 pixels. The angle of attack used for each airfoil ranges from -15° to +15° with a step of 1°. We obtain 40,165 images for the dataset. The angle of attack and lift-to-drag ratio have been scaled to between -1.0 and +1.0. Few samples of the airfoil images are presented in Figure 6.

**FIGURE 7:**
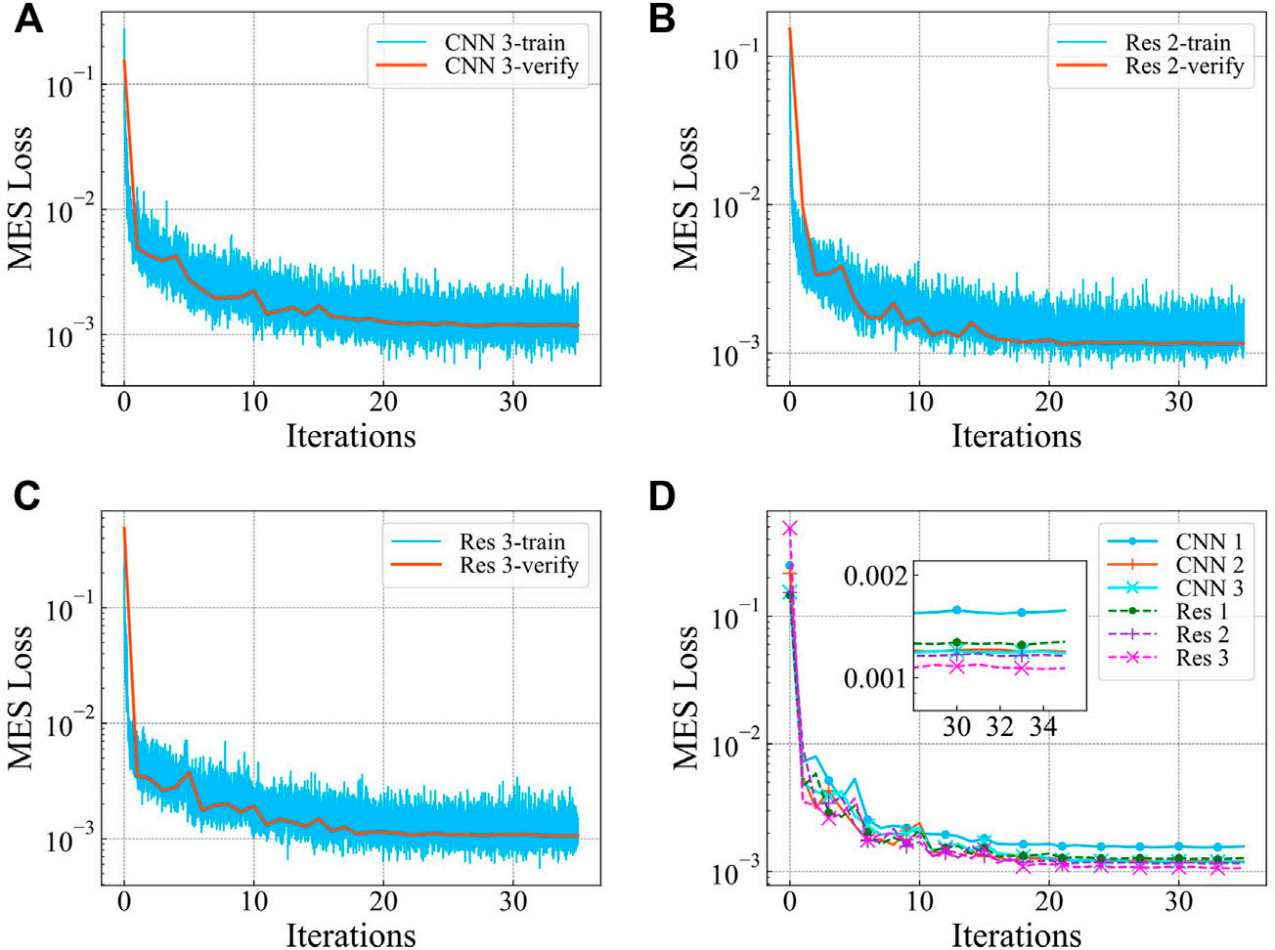
Loss function curve of network training **(A)** CNN Ⅲ **(B)** ResNet Ⅱ **(C)** ResNet Ⅲ **(D)** Validation set of all networks Loss function curve.

We use the Adam optimizer to optimize the weights of the network. According to the situation that the network occupies the video memory, we select a higher value for epochs, i.e., between 80 and 180. The initial learning rate is 0.001, and it is reduced by 70% every five iterations. When all the data in the training set has been used for training (once per iteration), we disable the dropout and use the validation set to calculate the value of loss function. During the training process, a total of 35 iterations are performed. We observe that the value of the loss function no longer decreases and remains stable. The loss function curve obtained using the verification set is shown in Figure 7.

### Lift-to-drag ratio prediction

We use the trained network to predict the lift-to-drag ratio using the samples from the validation set. The error of the prediction results is shown in Table 1. Among all the single networks, ResNet Ⅲ, ResNet Ⅱ, and CNN Ⅲ have the highest prediction accuracy. Therefore, the subsequent airfoil optimization work is based on these three networks.

**TABLE 1:** The error distribution of each neural network in the predicted value of the lift-to-drag ratio in the verification set.

Network	Less than 1%	Less than 5%	Less than 10%	Less than 15%
CNN Ⅰ	23.2	78.7	95.5	98.8
CNN Ⅱ	26.8	84.3	96.7	99.1
CNN Ⅲ	**31.3**	85.6	96.9	98.9
ResNet Ⅰ	26.6	83.6	96.6	99.0
ResNet Ⅱ	29.2	85.3	97.0	99.0
ResNet Ⅲ	29.6	85.3	97.0	**99.4**
Adjective (ResNet Ⅲ& ResNet Ⅱ)	30.4	**86.2**	**97.3**	99.3

The best results under each error level are shown in bold.

As a conventional deep learning algorithm, CNN has good prediction performance Yun et al. (2022); Sun et al. (2022). Based on the network architecture presented in Figure 4, it is evident that the predictive ability of the neural network is affected by the number of single-layer convolution channels and the number of layers. The network with more layers and channels has a stronger predictive ability. The ResNet Ⅲ and CNN Ⅲ have the same number of convolutional layers, number of channels, and similar architecture. There is no obvious difference between CNN Ⅲ and ResNet Ⅲ in predicting the lift-to-drag ratio. ResNet Ⅲ has a lower root mean square error considering the loss function value. In the detailed error distribution, there are more sample errors in the prediction results of CNN Ⅲ that are less than 1%. Contrary, the ResNet Ⅲ has more sample errors less than 15%. Therefore, the CNN Ⅲ achieves higher accuracy. But ResNet Ⅲ has better generalization performance. Similarly, the ResNet Ⅱ also achieves good prediction results. The predicted values of the two networks are weighted and averaged (the weight is 0.5) to improve the prediction accuracy of the network’s lift-to-drag ratio. The prediction accuracy after this combination is better than a single network within most of the error ranges.

**FIGURE 8:**
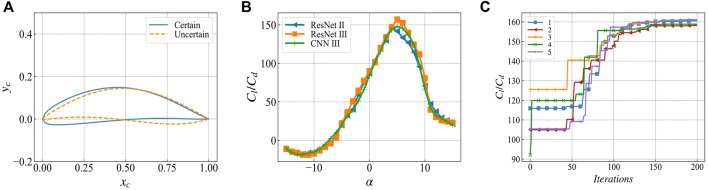
Geometry and lift-to-drag ratio prediction of the determined airfoil and the uncertain airfoil **(A)** solid line: the determined airfoil from the data set; dashed line: the uncertain airfoil generated by WGAN **(B)** different network pairs Determine the lift-to-drag ratio prediction of the airfoil, showing consistency **(C)** The lift-to-drag ratio predictions of the uncertain airfoil of different networks are significantly different.

Figure 8A shows the predicted value of the lift-to-drag ratio between the trusted airfoil and the unknown airfoil. Figure 8B shows that the predicted values of the lift-to-drag ratio for the credible airfoils of different networks are similar. Within the range of angle of attack, i.e., -15°–15°, the lift-to-drag ratio curves coincide, and the largest error occurs in the case of ResNetⅡ. In ResNetⅢ, the maximum error is 11.8% at 6° angle of attack. When the network output is uncertain, the predicted values of the three networks are very different. The error for ResNet Ⅱ and ResNet Ⅲ reaches 30.1% at an angle of attack of 8°. The error between ResNet Ⅲ and CNN Ⅲ is 8° and the angle error is 59.0%.

Based on the aforementioned results, we observe that:

(1) The uncertainty in network output is mainly concentrated in the range.
(2) In the range of the angle of attack, where the output uncertainty is obvious, there may be some points where the error is small, as shown in Figure 8C.

Ideally, the error sampling points are as dense as possible, i.e., In formula (8), the number of constraint points n of the lift-drag ratio prediction error should be as large as possible to ensure that the prediction error meets the requirements in the entire optimization range. However, at the same time, this increases the computational complexity. Considering comprehensively, in this work, we select an error limit area. At the same time, four angles of attacks are randomly selected for error control within the range of angle of attack by Latin super-sampling Mckay & Beckman (1979).

### Lift-to-drag ratio optimization

**FIGURE 9:**
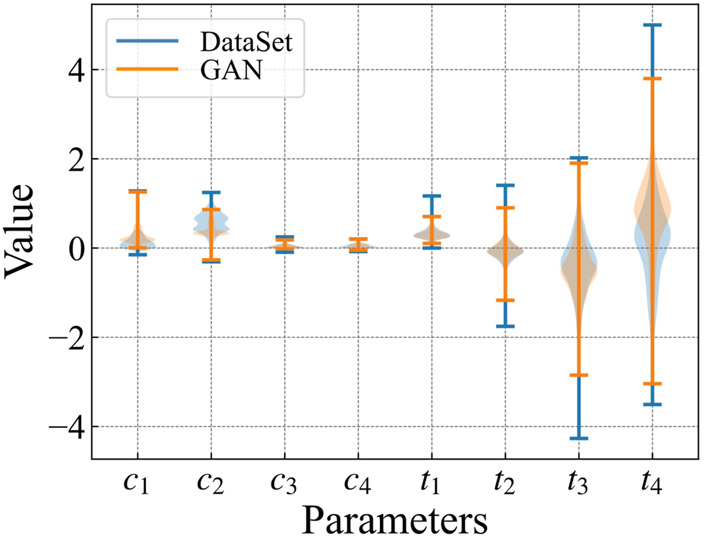
The IPG parameter distribution of the WGAN generated airfoil and the data set airfoil.

The lift-to-drag ratio optimization is based on randomly distributed particles. These particles are generated by using WGAN-GP. The distribution of the generated particles and the samples in the dataset are shown in Figure 9. The particle distribution of WGAN-GP is similar to the dataset.

During the optimization process of RDPSO-Gbest-R, the initial maximum particle velocity is 0.4. Consequently, the particles move randomly in the entire allowable optimization space and converge to the global optimal value. Afterwards, the maximum speed is halved after every 20 iterations so that the optimization results become gradually accurate. This principle is similar to the simulated annealing algorithm Bangert (2012).

(1) The influence of the number of particles on the optimization results

Machine learning algorithms are widely used in various fields of studies Tian et al. (2021); Xu et al. (2022); Yao et al. (2021). Optimization algorithms can boost predictive performance of machine learning Liu X. et al. (2022),Liu et al. (2022b); Zhao et al. (2022). In this work, we use the number of particles in RDPSO algorithm M = 50 and M = 100 to optimize the airfoil under the same optimization objective and compare the influence of the numbers of particles on optimization results. To avoid contingency, the airfoil optimization is repeated 5 times for each parameter. The optimization goal is the weighted average of the lift-to-drag ratio under the angle of attack (here, the weight coefficients are all), and formula (6) takes 0.5.

**FIGURE 10:**
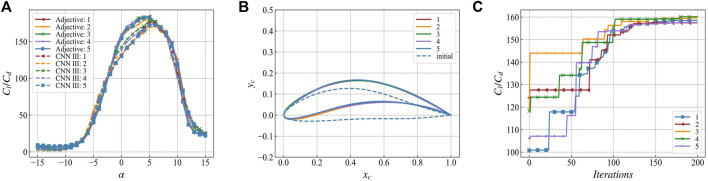
The optimization result of ion number M = 50 **(A)** The lift-drag ratio prediction of the five optimization results by Adjective and CNN Ⅲ **(B)** The airfoil geometry of the five optimization results and the initial airfoil optimized at one time **(C)** 5 times The change curve of the weighted value of the lift-to-drag ratio during the optimization process.

**FIGURE 11:**
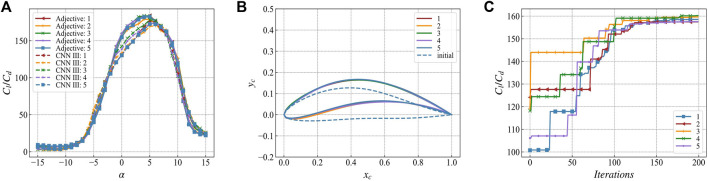
The optimization result of the number of ions M = 100 **(A)** The prediction of the lift-to-drag ratio of the fifth optimization result by Adjective and CNN Ⅲ **(B)** The airfoil geometry of the fifth optimization result And the initial airfoil of one of the optimizations **(C)** The weighted value change curve of the lift-drag ratio during the fifth optimization process.

Figures 10, 11 show the optimization results for M = 50 and M = 100, respectively. In the lift-to-drag ratio prediction of the optimization results, it is evident from Figures 10A, B that there is a difference between the multi-network joint prediction and the network CNN III that has not participated in the optimization. The optimization results are performed in the networks participating in optimization. This phenomenon is also evident in Figures 10A, B.

In the optimization results where M = 50, there are four results that converge to a consistent airfoil geometry. In the optimization results where M = 100, the lift-to-drag ratio curve, the airfoil geometry, and the weighted value of the lift-to-drag ratio converge to a consistent solution. In this case, the lift-to-drag ratio increases from 120 of the initial airfoil to at least 157, i.e., about 30.8%. GAN randomly generates the initial airfoil for each optimization, and finally a consistent optimization result is obtained. Therefore, this optimization result can be considered as the global optimal solution. The subsequent optimization calculations are performed with M = 100.

(2) Comparison of multi-network joint and single-network optimization results

**FIGURE 12:**
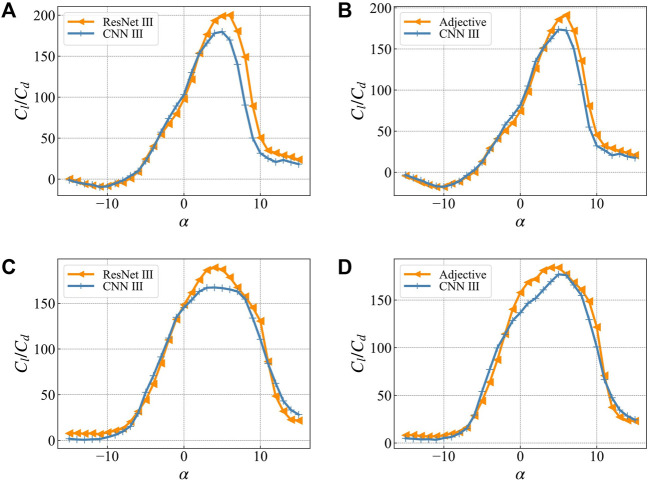
The optimization results are in the network participating in the optimization (ResNet Ⅲ or Adjective) and the network not participating in the optimization (CNN Ⅲ) The lift-to-drag ratio prediction curve in **(A)** is based on the optimization result of ResNet Ⅲ for single target angle of attack **(B)** is based on the optimization result of Adjective for single target angle of attack **(C)** is based on the optimization result of ResNet Ⅲ for multi-target angle of attack **(D)** Optimization results based on Adjective for multi-target angles of attack.

To verify the improvement in accuracy of the optimization results obtained by using multi-network collaboration, in this work, we use ResNet Ⅲ as it is the best performing network in lift-to-drag ratio prediction (Table 1; Figure 7D), and the multi-network collaborative adjective for a single attack. We optimize the angle (2°) and multiple angles of attack (same as the previous section). The number of particles and the optimization results of lift-to-drag ratio are shown in Figure 12. The triangles represent the predicted value of the lift-to-drag ratio of the network participating in the optimization based on ResNet Ⅲ or adjective. The "+" marks represent the predicted value of the lift-to-drag ratio of the independent network CNN III.

In the optimization result of a single angle of attack (Figure 12A), there is a significant difference between the single network predicted value and the actual value, and the maximum error reaches 32.7%. In contrast, the multi-network collaboration significantly reduces the difference between the predicted value of the optimized network and the verified network (Figure 12B). The maximum error in this case is 16.7%, which is 16.0% lower as compared to a single network. As shown in Table 2.

**TABLE 2:** Single network (ResNet Ⅲ) and multi-network collaboration (Adjective) optimize the angle of attack of single and multi-target. The maximum error between the predicted value of the lift-to-drag ratio of the network participating in the optimization and the predicted value of the result of the independent reference network CNN Ⅲ.

Optimization target	ResNet Ⅲ maximum error (%)	Adjective
		Maximum error (%)
Single target angle of attack	32.7	16.7
Multi-target angle of attack	13.0	12.3

While optimizing multiple angles of attack (Figure 12C), there is still a significant difference between the predicted value of the single network and the test network, i.e., the maximum error is 13.0%. The multi-network prediction results have a slightly lower error than a single-network prediction result, i.e., the maximum error is 12.7%, which is a decrease of 0.7%. This is because when the multi-angle attack optimization is performed, the output result may be too small or too large due to the uncertain output state of the network. In addition, the output of the small result makes the final weighted value lower than the certain value. To a certain extent, the shape of the optimal airfoil suppresses the influence of uncertainty during the optimization process.

(3) Comparison of optimization results with the best airfoil in the dataset

**FIGURE 13:**
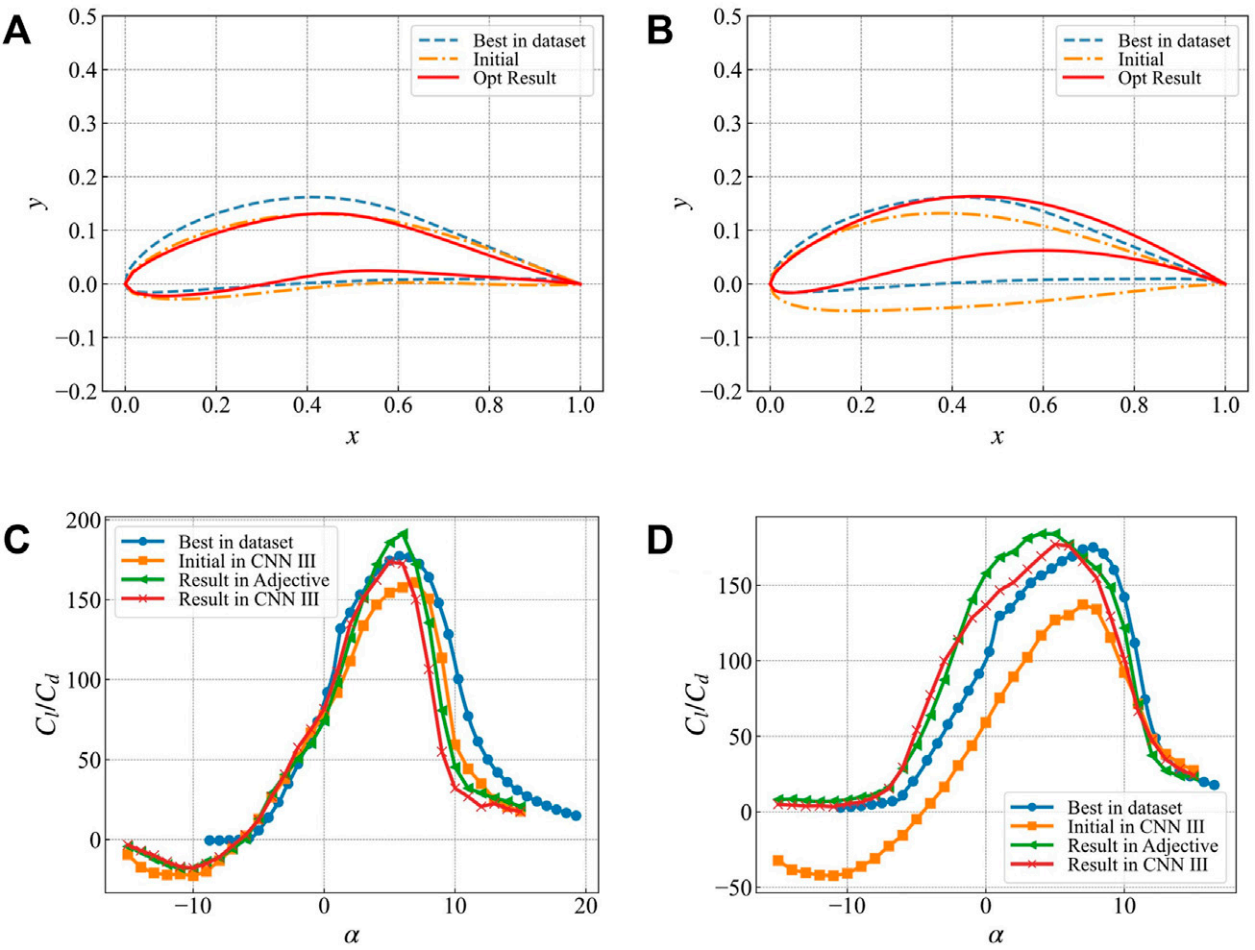
Comparison of optimization results with the best airfoil under the corresponding target in the data set **(A)** Airfoil geometry with 6° single angle of attack lift-to-drag ratio as the optimization target **(B)** The multi-angle of attack lift-drag ratio is the airfoil geometry under the target **(C)** The lift-drag ratio curve under the target with 6° single angle of attack lift-drag ratio **(D)** The lift-drag ratio curve under the target with multiple angles of attack lift-drag ratio.

Figure13 compares the optimization results of single angle of attack and multiple angles of attack targets. In addition, it also presents the comparison of the best airfoil geometry in the dataset for the corresponding target angles of attack. The optimization results are shown in Table 3. Figures 13A, C present the single angle of attack optimization results. The initial airfoil, optimal airfoil, and the optimization results are relatively similar, and the lift-to-drag ratio curve presented in Figure 13C also has the similar characteristics. There is an obvious peak at the target angle of attack at 6°, and the maximum lift-to-drag ratio reaches 172. The lift-to-drag ratio at other angles of attack degenerates rapidly, indicating that this optimization algorithm has obvious pertinence to the optimization target.

**TABLE 3:** The optimization results of multi-network coordination at multiple angles of attack and single angle of attack, compared with the best airfoil in the data set.

Optimization target	ResNet Ⅲ maximum error (%)	Adjective
		Maximum error (%)
Single target angle of attack	32.7	16.7
Multi-target angle of attack	13.0	12.3

Figures 13B, D present the optimization results of multiple angles of attack. For the optimization goal, the weighted value of lift-to-drag ratio after deep learning optimization is 147 (the optimal estimate is 159), which is equivalent to the best airfoil lift-to-drag ratio in the dataset. However, there is a big difference in the airfoil geometry. Both the optimization results and the best airfoils in the dataset maintain a high lift-to-drag ratio within a large angle of attack.

4) CFD-based lift-to-drag ratio verification and stall characteristic research We use the k-ε turbulence model of COMSOL Multiphysics to calculate the lift-to-drag ratio of the initial airfoil, the optimized airfoil, and the optimal airfoil in the database for verification. The calculation results are shown in Figure 13.

**FIGURE 14:**
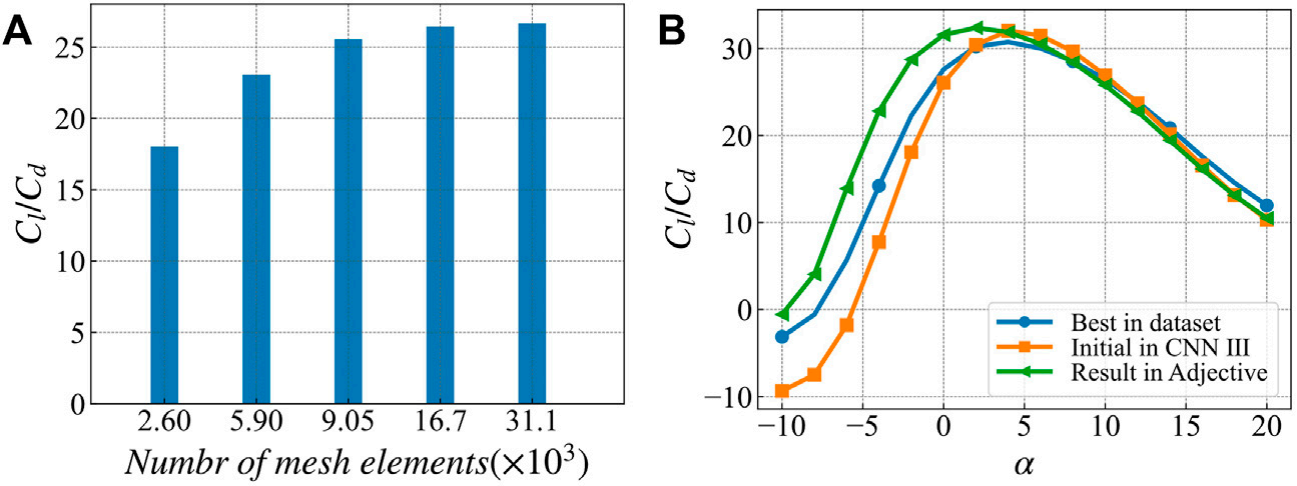
Verification of lift-drag ratio based on CFD optimization results. **(A)** Calculation results of lift drag ratio of the best airfoil in the database at 0° angle of attack with different grid numbers **(B)** lift drag ratio curves of the best airfoil, initial airfoil and optimized airfoil in the database.

Figure 14A shows the grid independence verification. The changing trend of lift-drag ratio is observed by changing the number of grid cells. When the number of grids is greater than 167,000, the calculation results no longer change significantly, indicating converging. Figure 14B shows the calculation results of the lift-drag ratio of each airfoil. The lift-drag ratio calculated by CFD is quite different from the value in the database. The reason is that the lift coefficient calculated by CFD is the same as the value in the database. In contrast, the value calculated by CFD The drag coefficient is larger, resulting in a significant difference in its ratio. The lift-drag ratio calculated in this paper is close to the airfoils in most other studies.

From the trend of the lift-drag ratio, the CFD calculation results are consistent with the prediction results of the neural network. The optimized airfoil has a lift-drag ratio similar to the best airfoil in the data set when the attack angle is greater than 5°, and the airfoil is less than 5° when the attack angle is less than 5°. The lower corners show a higher lift-to-drag ratio.

In the study of stall characteristics, we refer to the study of Guilmineau et al. (1997), using the SST k-ω model in COMSOL Multiphysics for the initial airfoil, the optimized airfoil, and the best airfoil in the data set. Stall characteristic studies were carried out. In the stall characteristic study, the airfoil rotates around 1/4 chord, and the angle of attack changes cyclically as follows:

$$\begin{array}{c} \alpha ={\text{α}}_{0}+{\text{Δ}}_{\text{α}}sin\left(2\text{πft}\right)=\alpha_{0}+{\text{Δ}}_{\text{α}}sin\left(2kt^{*}\right) \end{array}$$ (9)

Where $\alpha_{0}=5.0{}^{\circ}$ , ${\text{Δ}}_{\text{α}}=15{}^{\circ}$ , k=0.15 , $t^{\text{*}}=tU_{\infty }/c$ .

**FIGURE 15:**
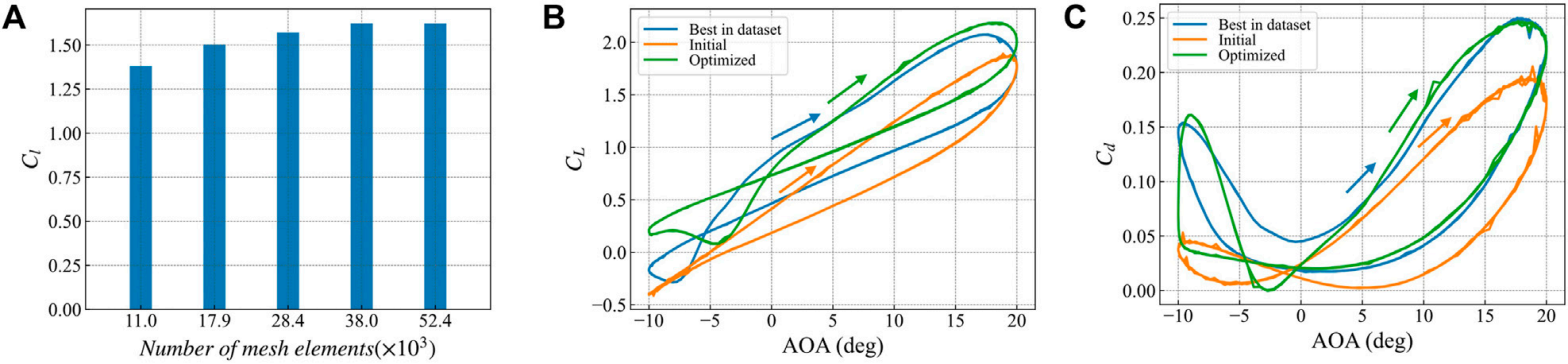
Stall characteristics of optimization results. **(A)** Calculation results of lift drag ratio of different grid numbers for the best airfoil in the database at 10° angle of attack **(B)** change curve of lift coefficient of the best airfoil, initial airfoil and optimized airfoil in the database with angle of attack **(C)** change curve of drag coefficient of the best airfoil, initial airfoil and optimized airfoil in the database with angle of attack.

Figure 15A Shows the grid independence verification. Different grid densities are used to calculate the lift coefficient at an angle of attack of 10°. The comparison shows that when the number of grids is greater than 38,000, the calculation results do not change significantly, indicating that the number of grids is sufficient. Stall characteristic calculations were performed using a grid with 38,000 elements. The lift coefficient and drag coefficient results are shown in Figures 15B, C, respectively.

The hydrodynamic prediction provides an important reference for the optimal design of the structureSu et al. (2021). In the pitch-up stage, when the angle of attack is greater than 5°, the lift coefficient of the optimized airfoil is slightly higher than that of the optimal airfoil in the data set, and the peak lift coefficient is also higher than that of the optimal airfoil in the data set (Figure 15B); for the drag coefficient, when the angle of attack is greater than 5°, the optimized airfoil is slightly higher than the optimal airfoil in the data set. The peak drag coefficient is basically consistent with the optimal airfoil in the data set (Figure 15C). During the descent, the lift coefficient of the optimized airfoil is significantly higher than that of the best airfoil in the data set. In contrast, the drag coefficient has no significant difference.

In general, the peak drag coefficient of the optimized airfoil is the same as that of the best airfoil in the data set, while the peak lift coefficient is improved to a certain extent. The optimized airfoil has a smaller lift coefficient hysteresis loop area and better stability.

## Conclusion

Both ResNet and traditional CNN can achieve high accuracy in lift-to-drag ratio prediction. These architectures are able to achieve more than 96% of the airfoil prediction error of less than 10%, and more than 85% of the airfoil prediction error of less than 5%. The number of layers and the number of convolution channels in a network significantly influence the prediction accuracy.

The multi-network coordination effectively suppresses the deviation of the optimization result from the actual value in the single-objective angle of attack optimization. The optimization results show that the maximum error after multi-network collaborative optimization is 16.7%, which is approximately half as compared to the maximum error of single-network optimization. In case multi-angle attack optimization, there is no significant difference.

Based on the airfoil optimization performed using RDPSO and deep learning, a high-performance airfoil with a gap of less than 5% from the best airfoil in the dataset is obtained in a conservative estimation. In the more optimistic estimation, the deep learning optimization results have a better performance as compared to the best airfoil in the dataset. For practical applications requiring higher reliability, the airfoil optimization based on deep learning may be more suitable for pre-optimization, providing a good initial airfoil for other optimizations with higher accuracy.

## Data Availability

Publicly available datasets were analyzed in this study. This data can be found here: http://www.airfoiltools.com.
